# DNA methylation profiling to predict overall survival risk in gastric cancer: development and validation of a nomogram to optimize clinical management

**DOI:** 10.7150/jca.44436

**Published:** 2020-04-27

**Authors:** Xianxiong Ma, Hengyu Chen, Guobin Wang, Lei Li, Kaixiong Tao

**Affiliations:** 1 Department of Gastrointestinal Surgery, Union Hospital, Tongji Medical College, Huazhong University of Science and Technology, Wuhan 430022, China.; 2 Department of Pancreatic Surgery, Union Hospital, Tongji Medical College, Huazhong University of Science and Technology, Wuhan 430022, China.; 3 NHC Key Laboratory of Hormones and Development, Tianjin Institute of Endocrinology, Tianjin Medical University Chu Hsien-I Memorial Hospital, Tianjin 300070, China.; 4 Department of Breast and Thyroid Surgery, Union Hospital, Tongji Medical College, Huazhong University of Science and Technology, Wuhan 430022, China.

**Keywords:** Signature, DNA methylation, gastric cancer, OS, Nomogram

## Abstract

DNA methylation has been reported to serve an important role in the carcinogenesis and development of gastric cancer. Our aim was to systematically develop an individualized prediction model of the survival risk combing clinical and methylation factors in gastric cancer. A univariate Cox proportional risk regression analysis was used to identify the prognosis-associated methylation sites based on the differentially expressed methylation sites between early and advanced gastric cancer group, then we applied least absolute shrinkage and selection operator (LASSO) Cox regression model to screen candidate methylation sites. Subsequently, multivariate Cox proportional risk regression analysis was conducted to identify predictive signature according to the candidate sites. Relative operating characteristic curve (ROC) analysis manifested that an 11-methylation signature exhibited great predictive efficiency for 1-, 3-, 5-year survival events. Patients in the low-risk group classified according to 11-methylation signature-based risk score yield significantly better survival than that in high-risk group. Moreover, Cox regression analysis combing methylation-based risk score and other clinical factors indicated that 11-methylation signature served as an independent risk factor. The predictive value of risk score was validated in the testing dataset. In addition, a nomogram was constructed and the ROC as well as calibration plots analysis demonstrated the good performance and clinical application of the nomogram. In conclusion, the result suggested the 11-DNA methylation signature may be potentially independent prognostic marker and functioned as a significant tool for guiding the clinical prediction of gastric cancer patients' overall survival.

## Introduction

The incidence of stomach cancer in 2018 ranked sixth globally with 1,033,701 new cases (5.7% of new cases with all cancer types) 1. However, patients' survival is poor as it is the second leading cause of cancer-related death worldwide (8.2%) 1. The pathological type of stomach cancer was overwhelmingly stomach adenocarcinoma (STAD, 90%), which consists of two major types of gastric adenocarcinoma (Lauren classification): intestinal type or diffuse type 2. According to the data released by National Cancer Institute, “approximately 0.9 percent of men and women will be diagnosed with stomach cancer at some point during their lifetime” 3. The prognosis and 5 year survival rates for stomach cancer remain poor 4.

Although surgery is the major approach for treating stomach cancer, radio- and chemo-therapy are important adjuvant therapies to eliminate tumor cells from the human body. 5- Fluorouracil (5-FU) and cisplatin have been extensively used to cure stomach cancers for several decades. 5-Fu can impede thymidylate synthase and build in both RNA and DNA, inducing DNA damage and cytotoxicity 5, 6, while cisplatin can combine the base on a strand of DNA, and prohibit the replication and transcription of DNA in tumor cells. Although different types of chemotherapy agents are deployed against several types of cancers, chemo-resistance to anticancer drugs reduces the effectiveness of chemotherapy. One well-known epigenetic alteration of patients with cancer or chemo-resistance is the methylation or demethylation of specific DNAs. Several methylation alterations in apoptotic genes have been identified and used as epigenetic biomarkers in determining either chemoresistance or chemosensitivity to anticancer drugs 7. For instance, high expression of bone morphogenetic protein 4 (BMP4) was found to be correlated with cisplatin resistance and worse prognosis in patients with gastric cancer (GC) 8. Methylation of the BMP4 promoter inhibited the expression of BMP4. Therefore, targeted genetic inhibition of BMP4 expression revealed promising target therapy to improve the prognosis of gastric cancer. One study also found that methylation of CDKN2A (p16) showed a significant correlation with longer survival in 38 patients of the 5-FU adjuvant chemotherapy group 6.

Accumulating evidence has shown that molecular signatures predicting clinical prognosis in diverse kinds of cancers 9-11. Wang and colleagues collected samples from 218 patients with gastric cancer and found a methylation of the promoter of MDGA2 (MAM domain containing glycosylphosphatidylinositol) anchored in cancer samples. Multivariate analysis also demonstrated that hypermethylation of MDGA2 predicted shortened survival in patients with gastric cancer 12. By analyzing the data from 492 cases with advanced gastric cancer, one study found that the demethylation of L1 and SAT-a was independent prognostic factors for shorter overall survival (OS) and relapse free survival time in patients with advanced gastric cancer 13.

Therefore, researches on methylation of DNAs are promising in revealing predictive biomarkers for treatments response and may help doctors provide individualized treatments and improve patients' survival time. Using DNA methylation as a predictor has a few advantages over other biomarkers. For instance, DNA methylation had a higher stability both in vivo and ex vivo 14, the requirement of a smaller amount of specimens to obtain enough DNA for analyzing methylation 15, and higher accuracy 16. Some study suggested that combinations of DNA methylation as predictors may yield higher sensitivity and specificity than individual DNA methylation 15. Therefore, intact-genome methylation profiles of cancer specimens from patients with gastric cancer in The Cancer Genome Atlas (TGGA) databases were analyzed and a predictive risk model for overall survival based on methylation of DNAs, was established and tested in our study.

## Materials and Methods

### DNA methylation data of STAD

The TCGA level 3 DNA methylation data of patients with STAD and their related clinical information was downloaded from TCGA database with TCGAbiolinks package 17. Only methylation data measured with illumina Human Methylation 450 BeadChip (illumina Inc, CA, USA) was included.We used β values to stand for all of the DNA methylation levels which were calculated with M/(M+ U+100). 'U' represented the signal from unmethylated beads and 'M' represented the signal from methylated beads at the targeted CpG site. Only the data containing patients for whom clinical survival information was available was identified to analyze the association between DNA methylation levels and the related survival in STAD. Overall, 382 specimens with 485,577 DNA methylation sites were included in this study. These 382 samples were divided into a training dataset (first 70%) and a testing dataset (remaining 30%) based on TCGA series number. The training dataset was used for determining and building a prognostic signature. While the testing dataset was applied for verifying the predictive performance of the signature. The LASSO^18^ was used to select the candidate methylation sites predicting the prognosis of patients given the virtues such as the method had a smaller mean error (MSE) than conventional approaches, handling the multicollinearity issues, selecting overall variable and coefficients shrink. A publicly available R package glmnet^19^ was employed to perform LASSO with 1000 iterations. A desired solution, that was, a set of methylation sites were used to construct a prediction model when the training dataset, test dataset, and overall dataset yielded a relatively high Area Under The Curve (AUC) simultaneously.

### Data processing, normalization and identification of differentially expressed methylation sites

It is necessary to filter and preprocess the data before building the prediction model. Methylation sites whose beta value was not available (NA) in greater than 10% of the total sample were removed from the analysis. For methylation sites whose NA data was less than 10% of the total sample, the NA data assumed by using impute.knn function from impute package 20. Finally, data normalization was performed by using betaqn function from wateRmelon package 21.

In addition, all the samples were divided into early gastric cancer group and advanced gastric cancer group. The standardized beta was transformed to M value based on the formulation: M=log(β/(1-β)). M value was used to eliminate the difference caused by different probes. Subsequently, M value was conducted to identify differentially expressed methylation sites between early and advanced group by using dmpFinder function from minfi package^22^.

### Gene sets enrichment analysis and protein-protein interaction analysis

Mapping a set of genes to the related biological annotation in the clusterProfiler package^23^ is an important foundation for the success of the gene functional analysis of any high-throughput data. Kyoto Encyclopedia of Genes and Genomes (KEGG) pathway analysis and Gene Ontology (GO) enrichment analysis were conducted with clusterProfiler package to analyze the genes corresponding to the differentially expressed methylation sites at the functional level. P<0.05 was considered as statistically significant.

The protein-protein interaction(PPI) was evaluated using search Tool for the Retrieval of Interacting Genes (STRING) 24 database online. STRING (version 11.0) t covers 24,584,628 proteins from 5090 organisms. The genes corresponding to the differentially expressed methylation sites were mapped to STRING to evaluate the interactive relationships among them, and we selected the experimentally confirmed interactions which achieved a combined score >0.4 as significant. Then we constructed PPI networks with the CytoScape^25^ software. The sub-network modules were screened by plug-in molecular complex detection (MCODE) in CytoScape. The screening criterias were as follows: MCODE score > 3 and node number >6. In addition, the function and pathway enrichment analysis in the sub-modules were also carried out. P < 0.05 was considered statistically significant.

### Construction and evaluation of prediction model and statistical analysis

All statistical analyses were performed based on the R statistical package (R version 3.6.1) except as otherwise noted. The univariate Cox proportional hazard analysis of the above screened differentially expressed methylation was performed to identify methylation sites that were significantly (P < 0.05) relevant to patient survival. Subsequently, the LASSO Cox regression analysis was performed to further select the candidate factors relevant to patient survival. After that, methylation sites were identified from the candidate signatures using as covariates to construct multivariate Cox proportional hazard model. Then, AUC was applied to assess the model value. The model with a better predictive performance was screened base on Akaike information criterion (AIC) value, the smaller the AIC, the better the model was. The prognostic risk scores for each patient were calculated by using the formula. The patients were then divided into high- or low-risk groups using the median risk score as the cutoff point. Then, the Kaplan-Meier (K-M) estimator with log-rank test (Mantel-Cox) was applied to test the cumulative survival time and evaluate the differences in OS between the two groups. Kaplan-Meier curves were drawn based on the “survival” package. Finally, the ROC analysis was performed using the “pROC” package 26 with a categorical variable for utility in predicting OS of patients. The greater the AUC was, the better the model was for the hazard prediction.

### Construction of the nomogram

A nomogram was performed based on the 'rms' R package. Factors that were used to construct the final multivariate Cox proportional hazard model were applied to conduct nomogram. C-index, ROC as well as calibration plots were performed to evaluate the prognostic value of the nomogram. The result of the nomogram was showed in the calibrate curve, and the 45° line implied the best prediction.

## Results

### Clinical characteristics of patients

The study was performed on 382 patients who were clinically and pathologically diagnosed with STAD. Of these patients, 246 (64.4%) were male and 136 (35.6%) were female. The median age at diagnosis was 67 years(range 30 years - 90 years) and the median overall survival time was 460 days. The pathologic stage was defined via the Cancer staging manual of American Joint Committee on Cancer. The stage of gastric cancer patients ranged from I to IV, with 50 (13.08%) patients in stage I, 127 (33.25%) patients in stage Ⅱ,172 (45.03%) patients in stage Ⅲ, and 33 (8.64%) patients in stage IV. The histological type derived from stomach and intestine was combined as unilateral cohorts for the analyse due to small numbers of sample. Histological type of the study patients included stomach adenocarcinoma (204 patients, 53.4%), stomach-intestinal adenocarcinoma (177 patients, 46.3%), and Discrepancy (1, 0.26%) , respectively. Twenty-two patients (5.76%) developed tumor metastasis and 344 (90.05%) patients did not. The metastasis status of the rest samples (16 patients, 4.19%) was unclear. Anatomic sites were located at a few positions of the patients' body, including Antrum/Distal, Cardia/Proximal, Fundus/Body, Gastroesophageal Junction, and other locations. Among which, Fundus/Body was the most common location (37.17%). The detailed clinicopathological characteristics of all the included patients are displayed in **Table 1**.

### Gene sets enrichment analysis and PPIanalysis

**Figure 1:**
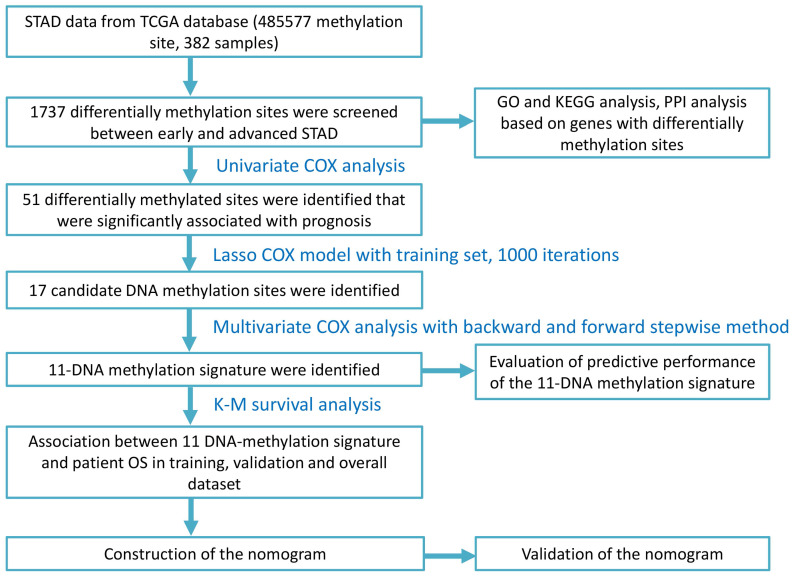
Flow chart of the present study.

In total, 1737 differential methylation sites were identified between early and advanced STAD groups based on differential analysis. **Figure 1** displayed the workflow of the present study.

**Figure 2:**
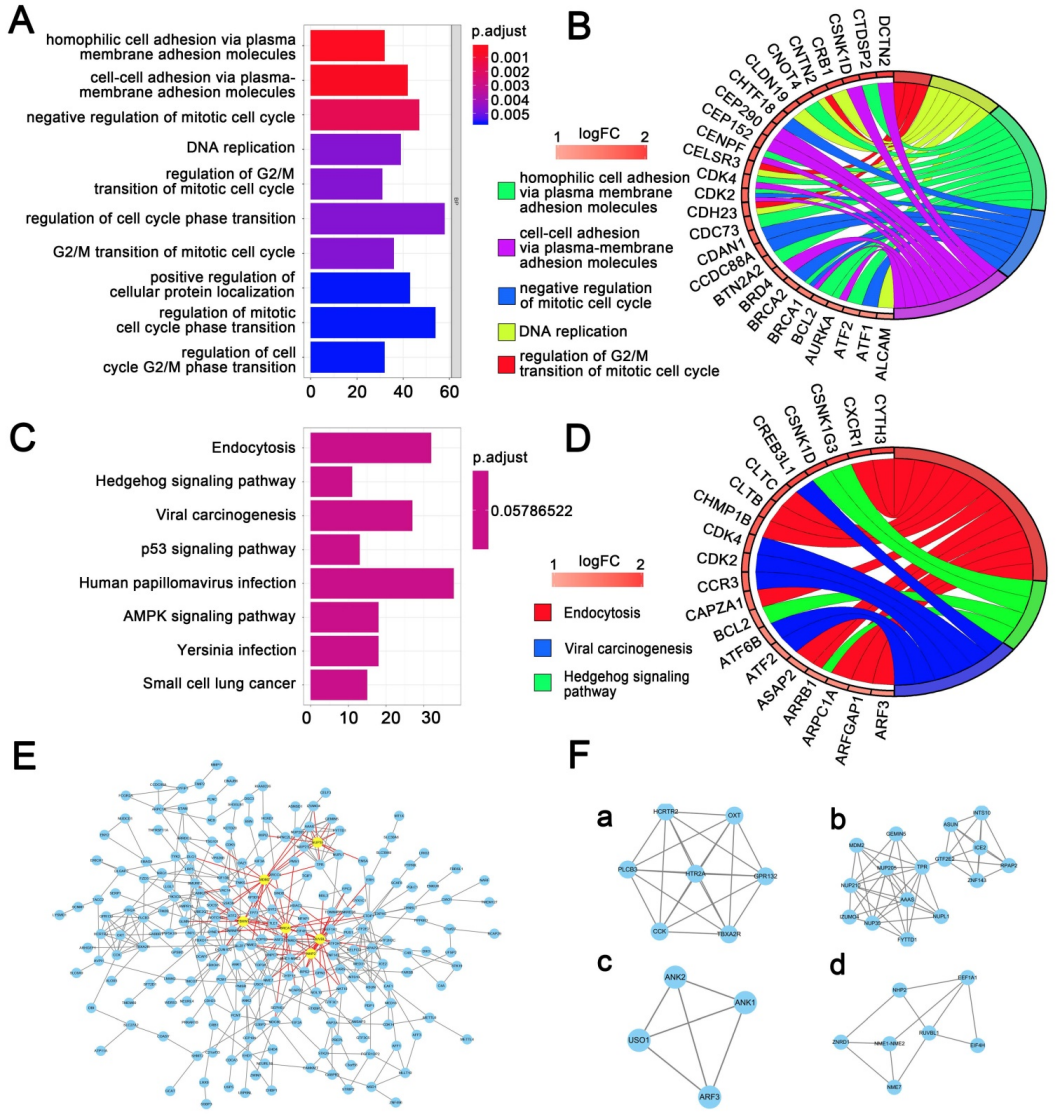
** Gene sets enrichment analysis and protein-protein interaction analysis. (A)** The top 10 GO enrichments for genes located in 1737 differentially expressed methylation sites. The original P value was transformed to '-log (P value)' in order to plot the bar chart. **(B)** The top 5 GO enrichments with its gene linkages. **(C)** The top 8 enriched KEGG pathway. **(D)** The top 3 KEGG pathway with its gene linkages. **(E)** Construction of protein-protein interaction network of genes corresponding to the 400 most significant differentially methylation sites. The big nodes represent the hub genes. **(F)** The top 4 sub-module from PPI network.

We conducted gene sets enrichment analysis for genes located at those differentially expressed methylation sites, which included GO analysis and KEGG pathway analysis. **Figure 2A and Figure 2C** displayed the top 10 enriched GO terms and significantly 8 enriched KEGG pathways, respectively. Accordingly, **Figure 2B and Figure 2D** presented the top GO terms, KEGG pathways and relevant genes. The top 3 enriched GO terms were homophilic cell adhesion through plasma membrane adhesion molecules, cell-cell adhesion by plasma-membrane adhesion molecules, and negative regulation of mitotic cell cycle **(Table S1)**. The top 3 enriched KEGG pathways were endocytosis, viral carcinogenesis, and hedgehog signaling pathway **(Table S2)**.

Besides, the genes corresponding to the 400 most significant differentially methylation sites were imported into the STRING database to build the interactive relationship between proteins. Only the genes with a combined score greater than 0.4 were selected to build the network. Finally, 458 pairs of protein relationships were identified after removing the unmatched genes. Genes with interactions more than 10 were considered as hub genes. Then 6 hub genes were identified: NHP2, NUP35, FBXW7, MDM2, BRCA1, RUVBL1 **(Figure 2E)**.

A total of 250 nodes and 458 edges were analyzed using plug-ins MCODE. The top 4 significant sub-modules were selected for the gene functional annotation. Enrichment analysis showed that the genes in those 4 sub-modules were mainly associated with G-protein coupled receptor protein signaling pathway, mRNA transport, establishment of protein localization, and GTP biosynthetic process **(Figure 2F)**.

### Identification of 11 methylation sites signature

**Figure 3:**
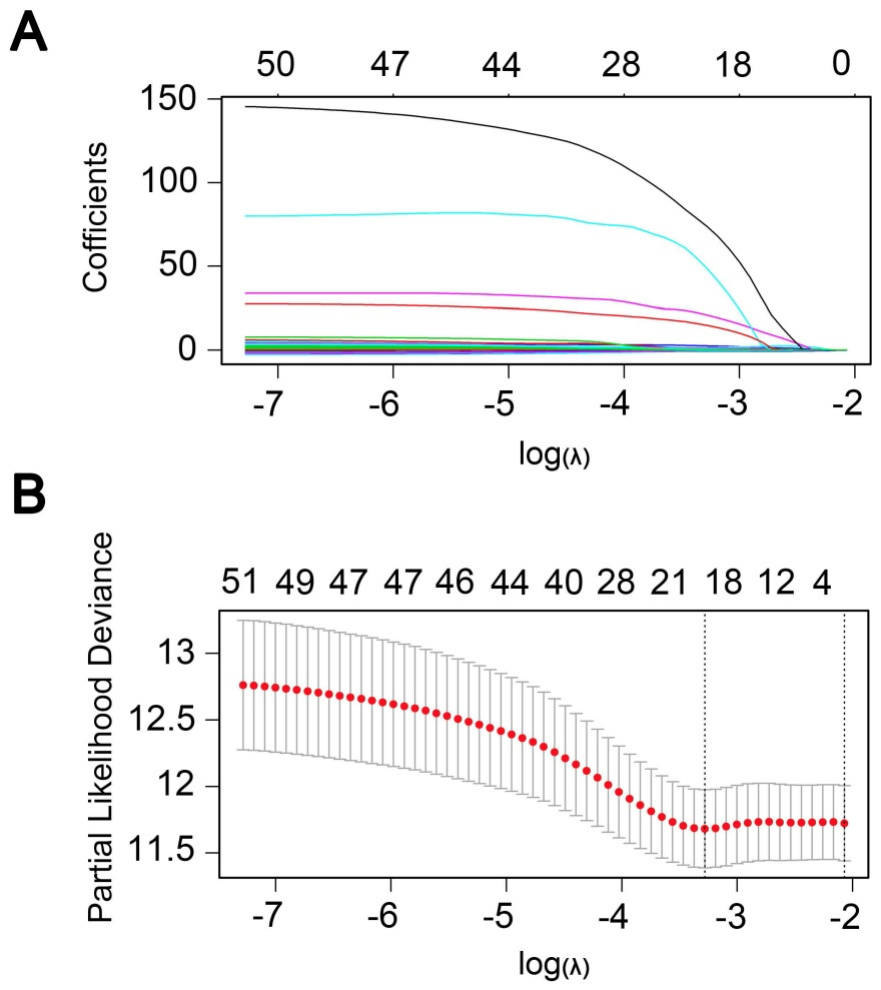
** Candidate methylation sites selection using the LASSO Cox regression model. (A)** 10-fold cross-validation for tuning parameter selection in the LASSO model via minimum criteria (the 1-SE criteria). **(B)** LASSO coefficient profiles of the 51 methylation sites. A coefficient profile plot was produced against log (lambda) sequence. Vertical line was drawn at the value selected using 10-fold cross-validation, where optimal lambda resulted in 17 non-zero coefficients.

**Figure 4:**
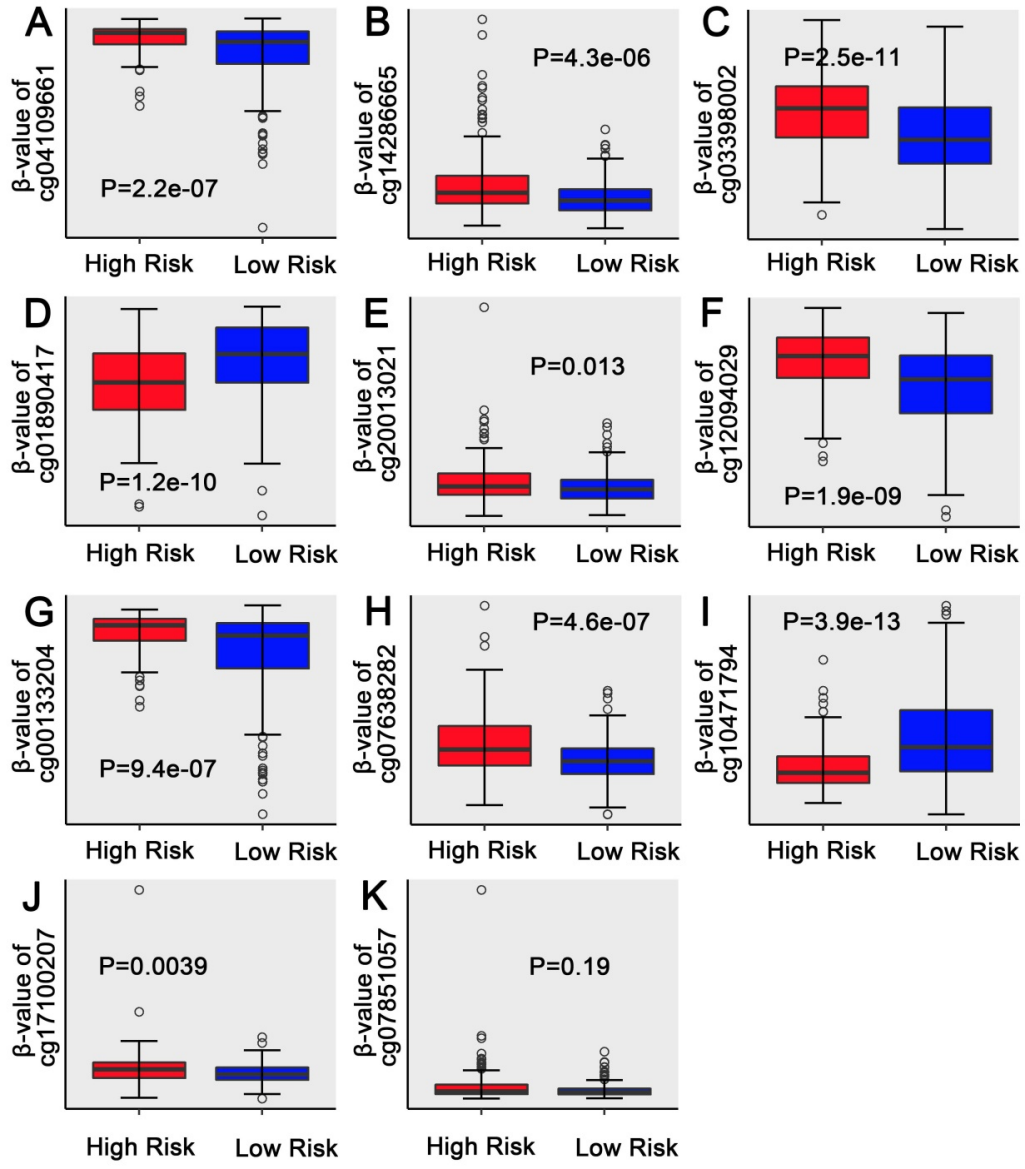
** Boxplots of methylation β values against risk group in the entire dataset.** “High Risk” and “Low Risk” represent the high-risk and low-risk group, respectively. The median risk score was taken as a cutoff. Y-axis represent the β-value of 11-DNA methylation sites respectively. The differences between the 2 groups were estimated by Mann-Whitney U test.

Univariate regression analysis of 1737 methylation sites showed that 51 methylation sites **(Table S3)** were significantly correlated with prognosis (p<0.01). The methylation expression profile of these 51 methylation sites was used to construct the LASSO Cox regression model and 17 methylation sites were identified as the candidate prognostic factors for predicting OS of patients. Of the 382 samples, 70% were randomly selected as training dataset, which was applied to build the model, and the other 30% as a testing dataset. When lambda was set as the lambda.min, the minimum mean cross test error was the minimum **(Figure 3A, 3B)**. Then, multivariate Cox proportional hazard regression model was constructed base on those 17 candidate methylation sites and a risk score formula of 11 methylation sites was identified finally: Risk score = 3.416*cg04109661 + 6.877*cg14286665 + 1.549*cg03398002 - 1.036*cg01890417 + 18.806*cg20013021 + 1.859*cg12094029 + 2.427*cg00133204 + 132.771*cg07638282 - 2.093*cg10471794 + 93.113*cg17100207 + 26.244*cg07851057. As a result, high-risk patients exhibited significantly higher methylation levels for cg04109661, cg14286665, cg03398002, cg20013021, cg12094029, cg00133204, cg07638282, cg17100207 and cg07851057, and significantly lower methylation levels for the other two methylation sites **(Figure 4D) (Figure S1-S2)**.

### Association between 11 DNA-methylation signature and STAD patients' OS in training, test and overall dataset

**Figure 5:**
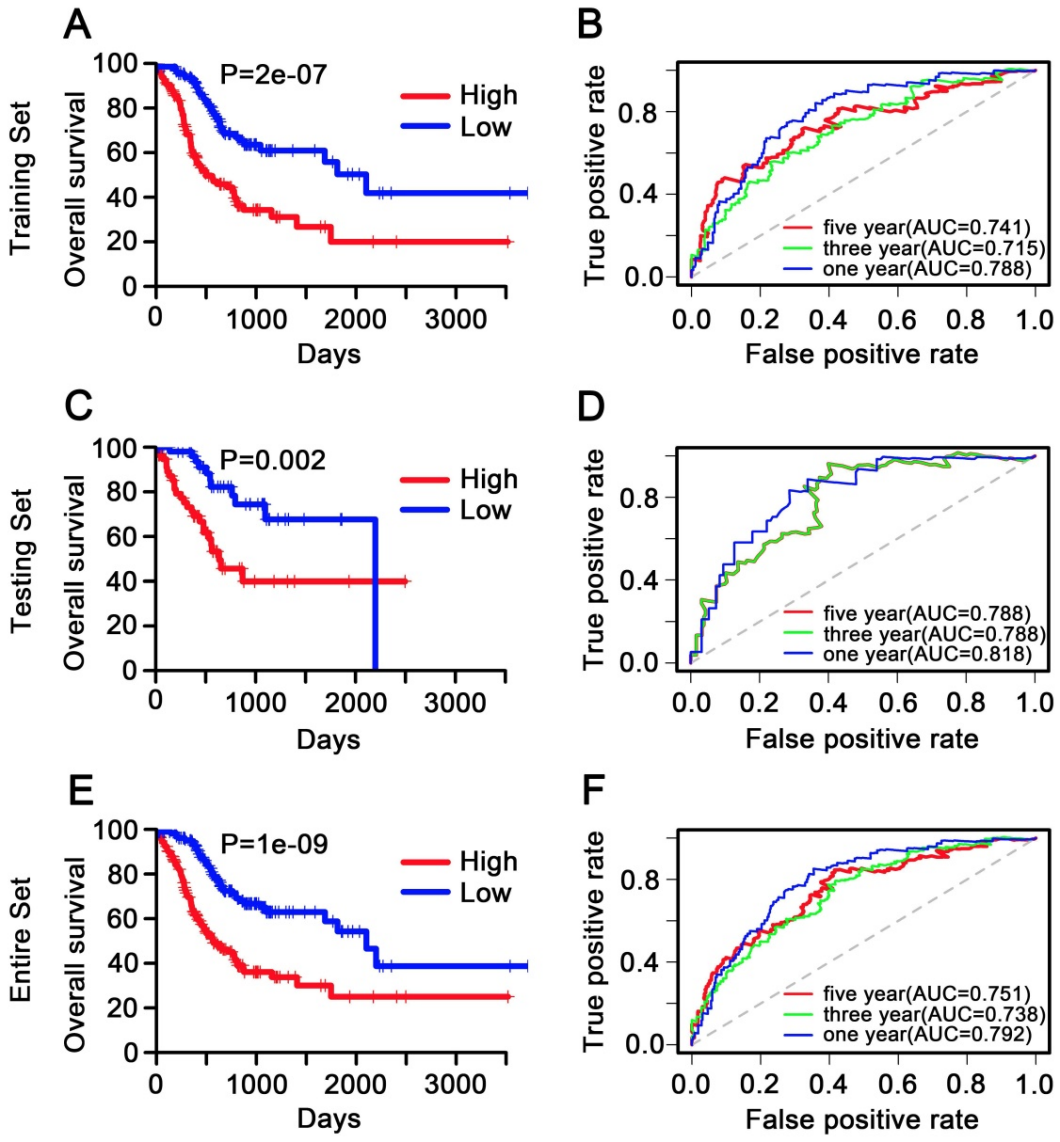
** Kaplan-Meier and ROC analysis of patients with STAD in training, testing and entire dataset, respectively. (A, C, E)** Kaplan-Meier analysis with two-sided log-rank test was performed to estimate the differences in OS between the low-risk and high-risk patients. **(B, D, F)** 1-, 3-, 5-year ROC curves of the 11-DNA methylation signature were used to demonstrate the sensitivity and specificity in predicting the OS of STAD patients. “High” and “Low” represent the high risk score group and low risk score group, respectively. The median risk score was taken as a cutoff.

The K-M analysis was performed in the training and testing datasets as well as the overall dataset to determine the underlying predictive value of the11-methylation signature in the overall survival. According to the median risk score, gastric cancer patients were divided into high-risk patients and low-risk patients. The median survival time of high-risk patients and low-risk patients was 383 and 574 days, respectively. As expected, patients with low risk score have better prognosis base on K-M survival curve in training dataset. Similar results were obtained in testing dataset and entire dataset. **(Figure 5A, 5C, 5E)**. These results confirmed that the 11-DNA methylation signature could stratify patients into high- and low-risk groups, implying its promising value in predicting STAD prognosis.

### Evaluation of predictive performance of the 11-DNA methylation signature

The AUC of ROC curve was used to assess the predictive accuracy of the 11-DNA methylation signature in training, testing, and entire dataset. The 5-year AUC of the 11-DNA methylation signature was 0.788, 0.741, 0.751, respectively **(Figure 5B, 5D, 5F)**, indicating that this model had high accuracy in predicting survival status in patients with STAD. This finding showed important significance in clinical gastric cancer treatment.

**Figure 6:**
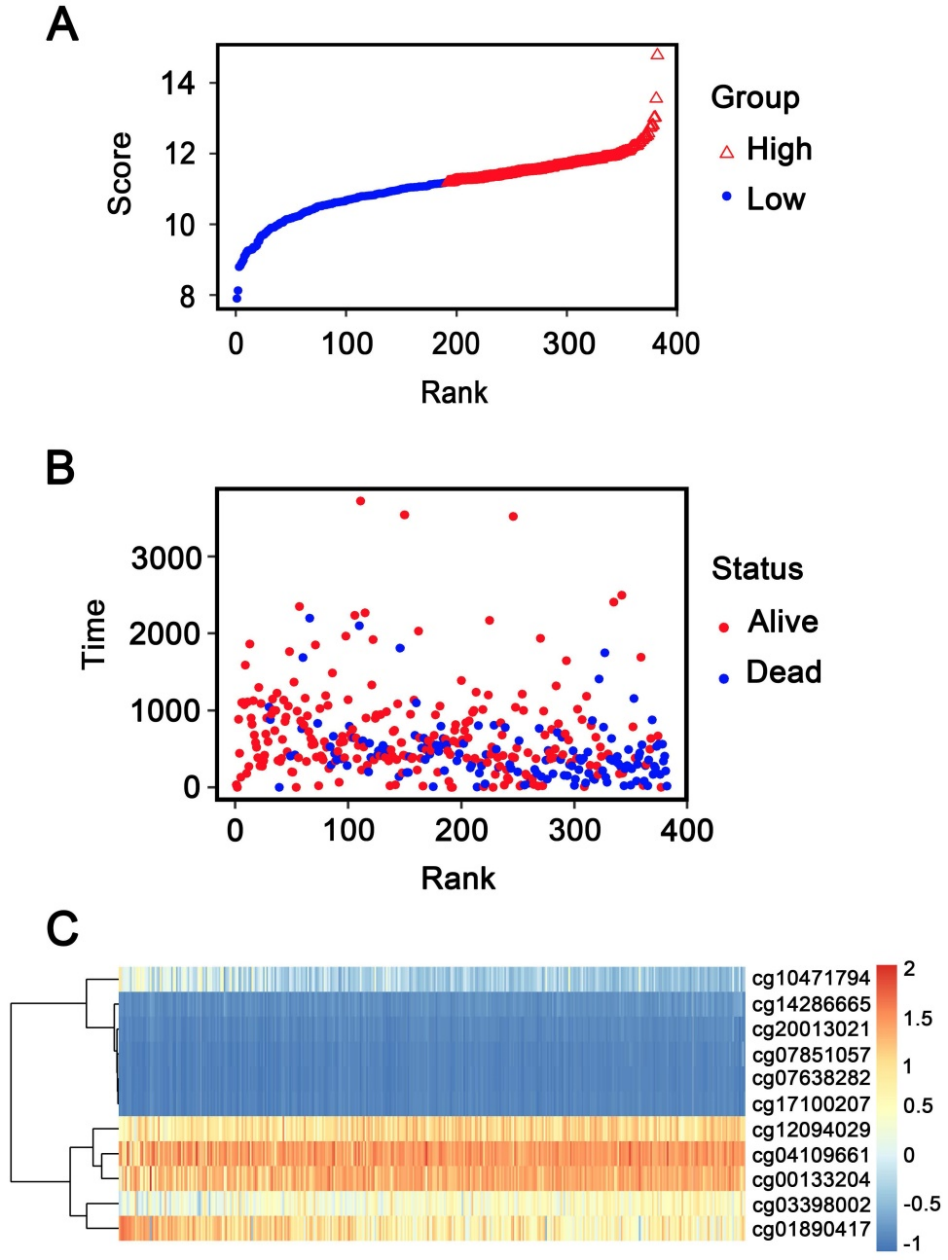
** Methylation risk score analysis of 382 GC patients in the entire dataset. (A)** Methylation risk score distribution against the rank of risk score. Median risk score is the cut-off point.** (B)** Survival status of GC patients. **(C)** Heatmap of 11 methylation sites expression profiles of GC patients.

In addition, patients were ranked according to their risk scores (**Figure 6A**), and the dot plot was drew according to their survival status (**Figure 6B**). Result indicated that the low-risk group had a lower mortality rate than the high-risk group. Heatmap of 11 methylation sites sorted by risk score were presented in **Figure 6C**, which was consistent with our previous boxplot.

Besides, Subgroup analysis was performed by various clinicopathological factors including gender, age, stage, pathological type, metastasis status, anatomic site, which also yielded a relatively high performance in most of sub-groups **(Figure S3-S8).**

### Nomogram development

**Figure 7:**
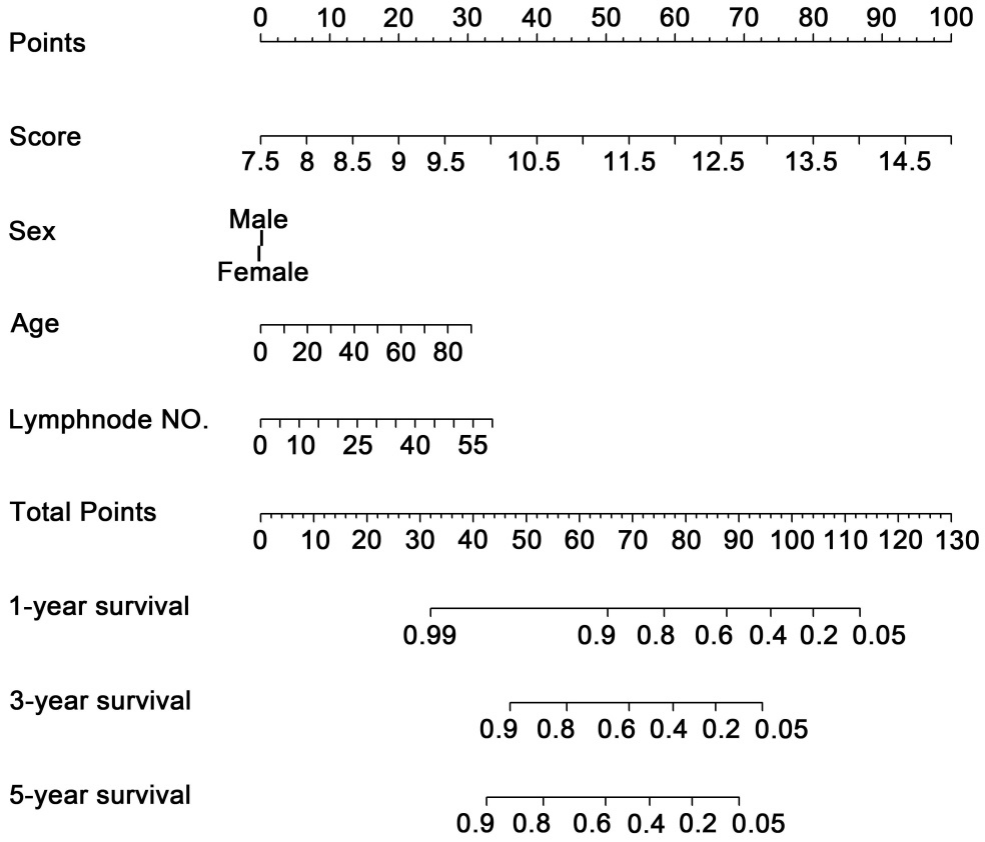
** Methylation nomogram for the prediction of GC's OS.** The nomogram was developed in the entire cohort, with the methylation risk score, sex, age, and positive lymph node numbers.

**Figure 8:**
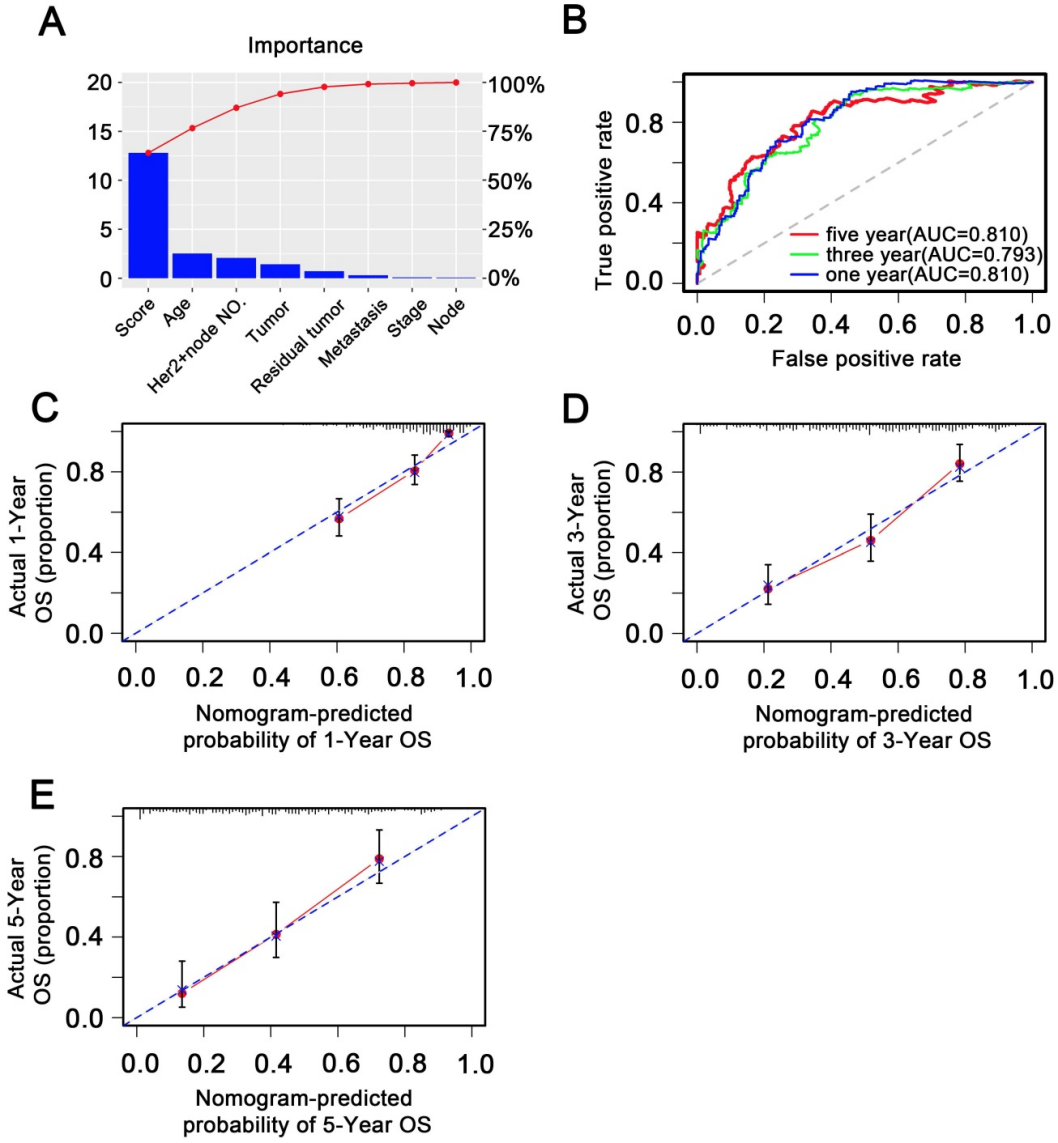
** Validation of methylation nomogram in the entire dataset. (A)** Barplot of importance of each clinical factor**. (B)** 1-, 3-, 5-year ROC curves for the methylation nomogram. **(C, D, E)** represent the 1-, 3-, 5-year nomogram calibration curves, respectively. The closer the dotted line fit is to the ideal line, the better the predictive accuracy of the nomogram is.

To investigate whether the 11-DNA methylation signature was an independent prognostic predictor of patient OS, univariate and multivariate Cox model was conducted on methylation associated risk score and several other clinicopathological factors. Hazard ratios (HRs) suggested that the 11-DNA methylation signature was significantly relevant to the OS of patients (P<0.001, HR 2.55, 95% CI 1.99-3.27) by the outcome of Cox regression analysis (**Table 2**), suggesting that the 11-DNA methylation signature was an independent prognostic predictor. To predict the prognosis of patients with STAD based on a quantitative method, we developed a nomogram (**Figure 7**) that combined both the 11-DNA methylation signature and the conventional clinicopathological factors which yielded significant P value in multivariate Cox model to predict OS. The importance of each factor was displayed in **Figure 8A**. The evaluative indicator such as C-index (0.760, 95%CI: 0.722-0.797), AUC (1-, 3-, 5- year: 0.810, 0.793, 0.810) (**Figure 8B**) and calibration plot yielded a high value simultaneously (**Figure 8C, 8D, 8E**).

The above data showed that the 11-DNA methylation signature could offer a better reference for different regrouped cohorts because of the effectiveness of risk stratification. The results indicated that the 11-DNA methylation signature showed better applicability when patients were regrouped based on different clinicopathological characteristics, suggesting that the 11-DNA methylation signature was an independent prognostic predictor of STAD patients survival.

## Discussion

The expression of specific DNAs has been shown a promising value in detecting the gastric cancer and predicting survival in patients with gastric cancer. A previous study reported that DNA hypomethylation was relevant to the progression of carcinoma and might contribute to chromosomal instability and genome rearrangement 27. In addition, It has been reported that the methylation status of particular genes was associated with a worse prognosis 28, 29, suggesting that alterations of methylation status may be related to the cancerous development. One study also used the methylation of specific DNAs to detect gastric cancer. The study investigated 51 candidate genes from 7 gastric cancer cell lines. They examined methylation status of these genes in a training population, which included 131 gastric neoplasia, then validated their findings in a test population consisting of 40 primary gastric cancer samples. The study found that six genes (MINT25, RORA, GDNF, ADAM23, PRDM5, MLF1) presented differential methylation between gastric cancer and normal mucosa in the training and test population. Among the six genes, MINT25 methylation was a sensitive (90%) and specific (96%) marker for screening in gastric cancer 30.

Tanaka Tomokazu and colleagues found that low expression of Trefoil factor 1 (TFF1) was relevant to poor survival in gastric cancer patients who were treated by surgery alone, while the expression of TFF1 was silenced by DNA methylation and was also associated with tumor invasion 31. Other studies also showed that high methylation status of IGF2 and LINE1 was associated with invasion of gastric cancer 32 and hypermethylation of GFRA3 promoter may predict poor overall survival in gastric cancer patients who underwent surgery 33. Besides, Li et al. reported that integrative analysis of DNA methylation and gene expression identified a six epigenetic driver signature for predicting prognosis in hepatocellular carcinoma 34. Nassiri revealed a DNA methylation profiling to predict recurrence risk in meningioma 35. All of above studies suggested that DNA methylation models displayed good performance for predicting prognosis for cancers, which have promising clinical application value.

By analyzing gene sites in 382 samples, we found that the methylation of 11-DNA was associated with prognosis in patients with STAD. Higher levels of 2-DNA methylation (ZNF644, cg10471794) were associated with better survival, while, higher level of 13 DNA methylation was associated with worse survival (MGC16121, KIAA1026, FBXO11, BDP1, CCDC126, XPO7, STX2, IPO5, cg12094029) in patients with STAD. A risk prediction model constituted by the 17 DNAs was established and showed good accuracy in evaluating the overall survival of patients.

A series of researches have revealed that the methylation of DNAs involved in risk prediction model was associated with cancer development and prognosis. One study found that the expression of ZNF644 was changed in patients with peripheral blood leukocytes who underwent chemotherapy 36. The expression of KIAA1026 was down-regulated in breast tumors and metastases 37. FBXO11 is a member of the F-box protein family. Previous studies revealed inconsistent findings on the role of FBXO11 in the process of cancerogenesis and in predicting cancer survival. Three studies showed that higher expression of FBOX11 was independent predictors of poor OS of patients with different types of cancer such as renal cell carcinoma, breast cancer, and hepatocellular carcinoma 38-40. However, other studies showed that FBXO11 acted as a tumor suppressor and higher expression of FBXO11 was associated with better patient survival 41-43. Our study found that methylation of FBOX11 was associated with worse survival which supported that FBXO11 acted as a tumor suppressor. Syntaxin2 (STX2) was associated with colorectal cancer (CRC) invasion and metastasis, as well as poor patient survival. The potential mechanism may be that STX2 may activate the nuclear transcription factor-κB (NF-κB) signaling pathway, and in turn, NF-κB increased STX2 expression. The positive signaling loop eventually enhanced CRC metastasis 44. IPO5, one of the karyopherin nuclear transport receptor family members, could contribute to the development of CRC by bounding to the NLS sequence and mediating RASAL2 nuclear translocation 45.

Apart from the desired results, limitations still existed in the present study. Firstly, the methylation data was divided into training set and internal validation set, lacking of external validation set, which may lead to some kinds of biases. Secondly, quite a long time was required for applying it clinically due to a high methylation testing charge. Despite the limitations mentioned above, there were still various valuable implications. In the present study, a 11-DNA methylation signature was established and could separate GC patients into two groups and predict OS with robust performance in not only training, testing and entire datasets but also in most of the sub-groups. Besides, we employed LASSO method to filter variables between univariate and multivariate Cox analysis, which perfectly solved the multicollinearity problem and made our results more reliable. Furthermore, few previous studies have combined methylation signature with clinical indicators to predict OS and no study was performed as above for gastric cancer yet. Our work demonstrated the transformative utility of integrating clinical and molecular factors for use beyond simple classification into the realm of individualized prognostication for GC and determined an individualized probability of OS for patients with GC, which represents a major advance in the field of personalized medicine for gastroenterology.

Although the clinical value of these 11 DNA methylation sites still remained to be fully investigated, those methylation sites had crucial connection with the prognosis of patients with STAD and may be an underlying therapeutic target for STAD. In addition, we developed a nomogram that combined both the 11-DNA methylation signature and the conventional clinicopathological factors to predict 1-, 3- and 5-year OS. The results might contribute to the development of effective molecular markers in the clinical routine.

## Conclusion

Therefore, the methylation of the 11-DNA signature may potentially be used as a novel independent prognostic biomarker to predict the OS of patients with gastric cancer. Further clinical studies on the functional mechanism of the 11 DNA methylation signature should be explored for the possibility of its involvement in the carcinogenesis.

## Supplementary Material

Supplementary figures.

Supplementary table 1.

Supplementary table 2.

Supplementary table 3.

## Figures and Tables

**Table 1:** Clinical characteristics of included patients with gastric cancer

Characteristics	Total	Training dataset (n=268)	Testing dataset (n=114)
Sex			
Female	136(35.6)	97(36.19)	39(34.21)
Male	246(64.4)	171(63.81)	75(65.79)
Histological type			
Stomach Adenocarcinoma	204(53.4)	139(51.9)	65(57.0)
Stomach- Intestinal Adenocarcinoma	177(46.3)	128(47.8)	49(43.0)
Other	1(0.26)	1(0.37)	
Stage			
Stage I	50(13.1)	30(11.2)	20(17.6)
Stage II	127(33.2)	94(35.1)	33(28.9)
Stage III	172(45.0)	121(45.1)	51(44.7)
Stage IV	33(8.64)	23(8.58)	10(8.77)
Metastasis status			
M0	344(90.05)	243(90.67)	101(88.6)
M1	22(5.76)	13(4.85)	9(7.89)
MX	16(4.19)	12(4.48)	4(3.51)
Age			
<=65	178(46.6)	120(44.78)	58(50.88)
>65	201(52.62)	145(54.1)	56(49.12)
NA	3(0.79)	3(1.12)	
Anatomic sites			
Antrum/Distal	141(36.91)	104(38.81)	37(32.46)
Cardia/Proximal	52(13.61)	34(12.69)	18(15.79)
Fundus/Body	142(37.17)	101(37.69)	41(35.96)
Gastroesophageal Junction	38(9.95)	20(7.46)	18(15.79)
Other	9(2.3)	9(3.3)	

**Table 2:** Univariate Cox regression analysis and multivariate Cox regression analysis outcome based on methylation risk score and other clinical factors.

	Univariate Cox analysis				Multivariate Cox analysis			
Characteristics	HR	HR.95L	HR.95H	P value	HR	HR.95L	HR.95H	P value
Score	2.718282167	2.16171357	3.418148473	1.18E-17	2.552291593	1.990295974	3.272976713	1.54E-13
Sex	1.21780532	0.857126589	1.730257605	0.27149398				
Age at initial pathologic diagnosis	1.022889192	1.007768508	1.038236749	0.002897625	1.026042322	1.008775545	1.043604647	0.002987916
Histological type	1.096262962	0.788648413	1.523863438	0.584410769				
Tumor	1.376148414	1.109495583	1.706887784	0.003666749	1.35521211	1.016922667	1.806036903	0.03803348
Lymph node status	1.362646347	1.178750091	1.575232171	2.87E-05	0.977914047	0.76106433	1.256550656	0.861394092
Metastasis status	1.418931078	1.052366548	1.913178833	0.021750423	1.128545617	0.792286856	1.607517783	0.502861885
Stage	1.60885934	1.302363986	1.987484608	1.03E-05	0.958739204	0.653299624	1.406982076	0.82953355
Grade	1.154911618	0.974517914	1.368698127	0.096496554				
Pylori infection	0.963926203	0.737620758	1.2596632	0.787844008				
Reflux history	0.924983022	0.724883645	1.180318519	0.530665644				
Residual tumor	1.63205323	1.328798901	2.004515313	3.01E-06	1.17339795	0.922264043	1.492916004	0.193131887
Anatomic neoplasm subdivision	0.951290227	0.841094269	1.075923507	0.426631743				
Ethnicity	1.019005844	0.741051981	1.401214674	0.907767476				
Race	1.084867725	0.885392834	1.329283382	0.432000455				
Number of lymph nodes positive	1.048322314	1.032019933	1.064882217	3.60E-09	1.033726554	1.008532457	1.059550024	0.008417234

## Abbreviations

OS: overall survival
GC: gastric cancer
STAD: stomach adenocarcinoma
LASSO: least absolute shrinkage and selection operator
5-FU: 5-fluorouracil
TGGA: The Cancer Genome Atlas
MSE: smaller mean error
NA: not available
PPI: protein-protein interaction
MCODE: molecular complex detection
K-M: Kaplan-Meier
HRs: Hazard ratios
CRC: colorectal cancer
AIC: Akaike information criterion
AUC: Area Under The Curve
RFS: relapse free survival
ROC: Relative operating characteristic curve
KEGG: Kyoto Encyclopedia of Genes and Genomes
GO: Gene Ontology
