# Supplementary material for: DNA methylation profiling to predict overall survival risk in gastric cancer: development and validation of a nomogram to optimize clinical management
Source: J Cancer. 2020 Apr 27;11(15):4352–65. doi: 10.7150/jca.44436 (PMC7255367; doi:10.7150/jca.44436)
Supplement: Supplementary file 1 — Supplementary figures. [file jcav11p4352s1.pdf]

Supplementary Figure S1

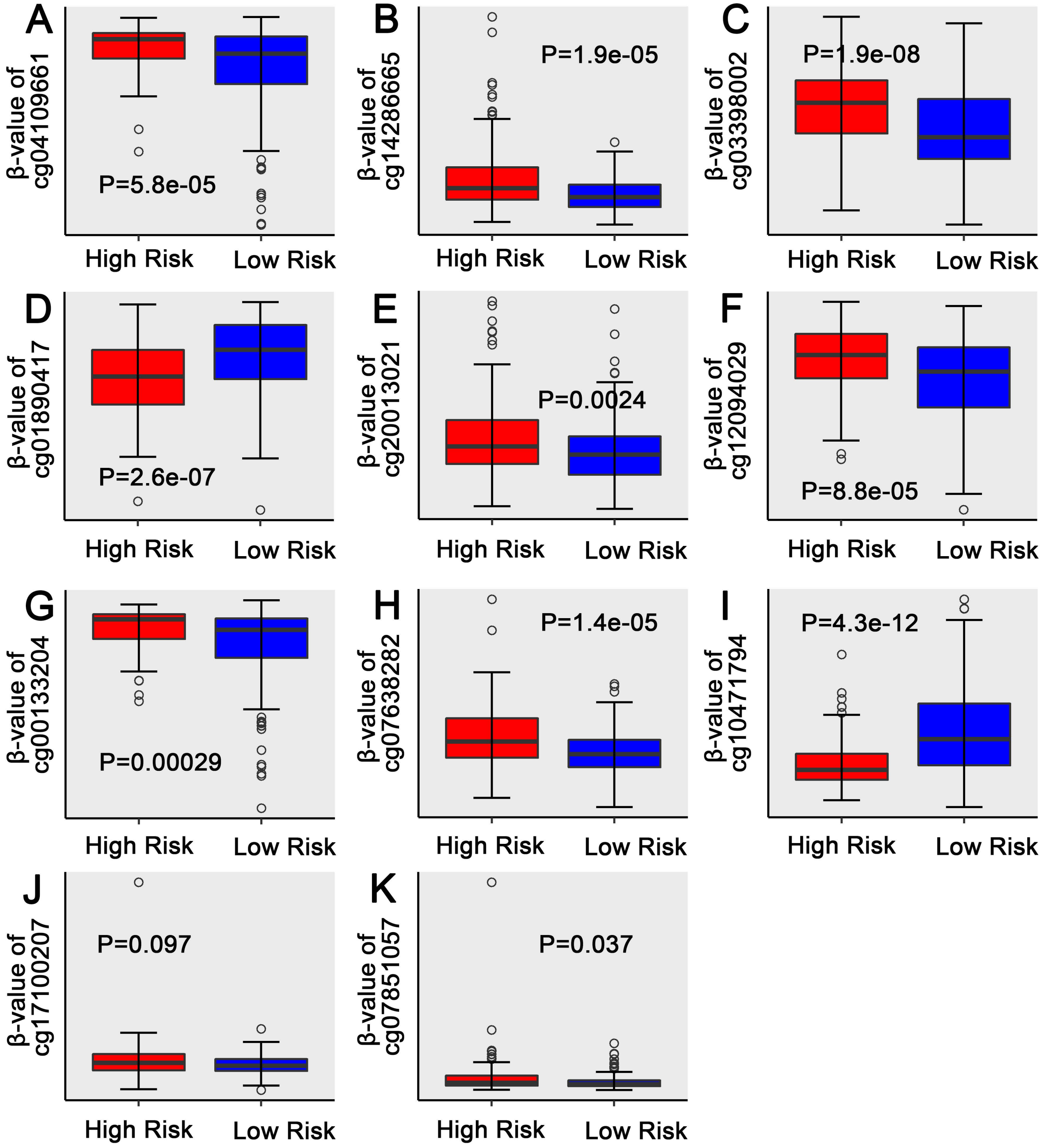

Supplementary Figure S2

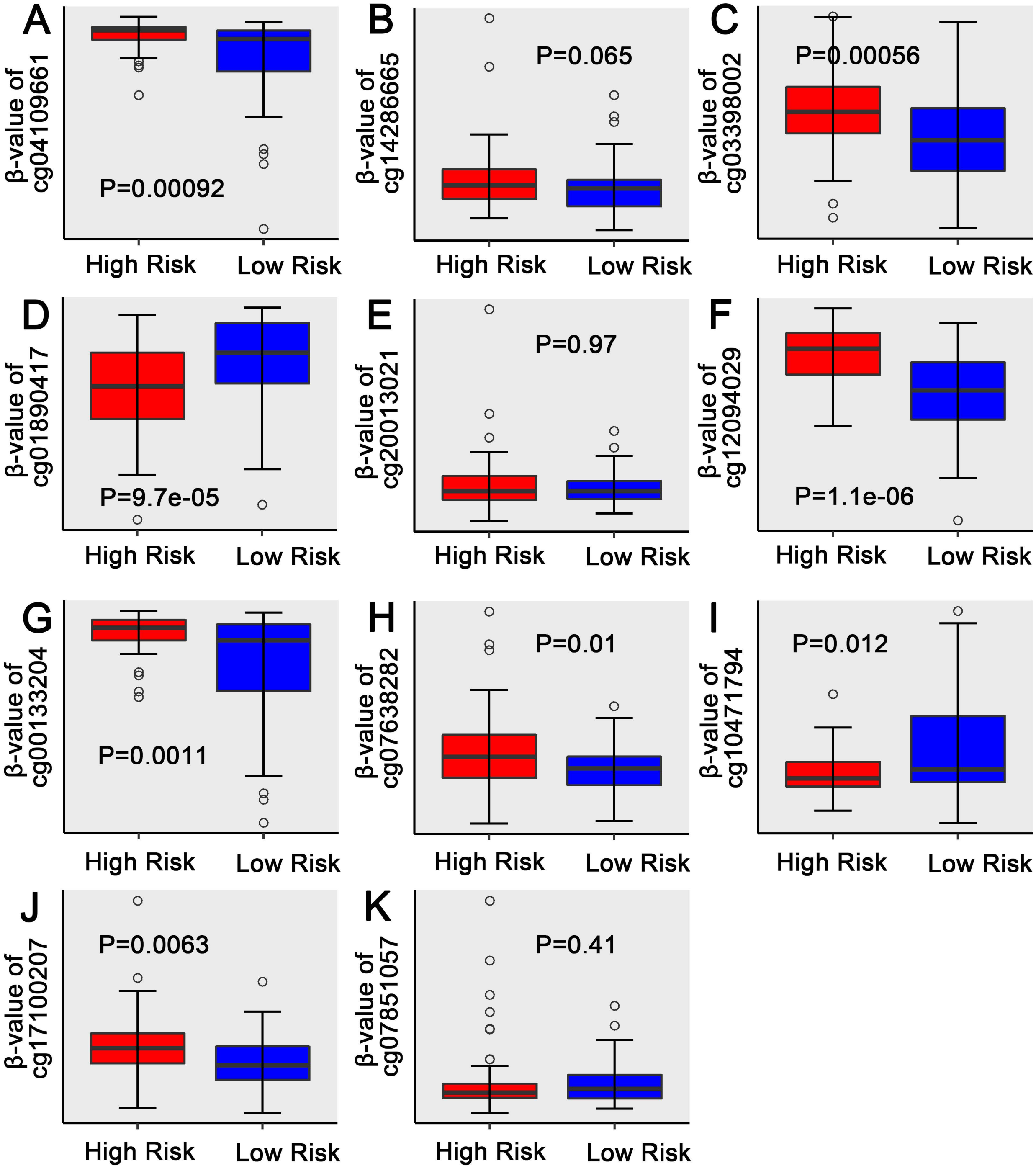

Supplementary Figure S3

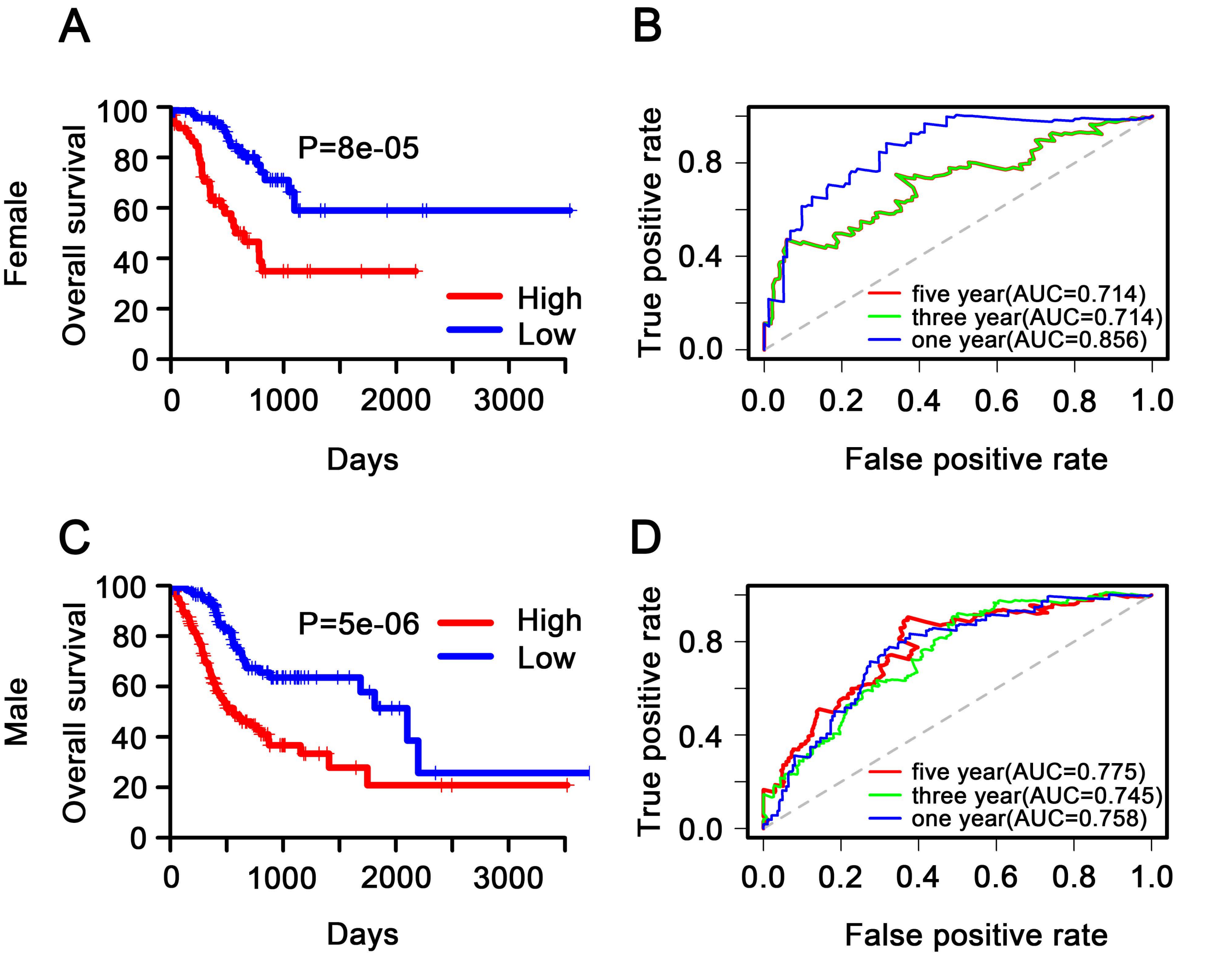

Supplementary Figure S4

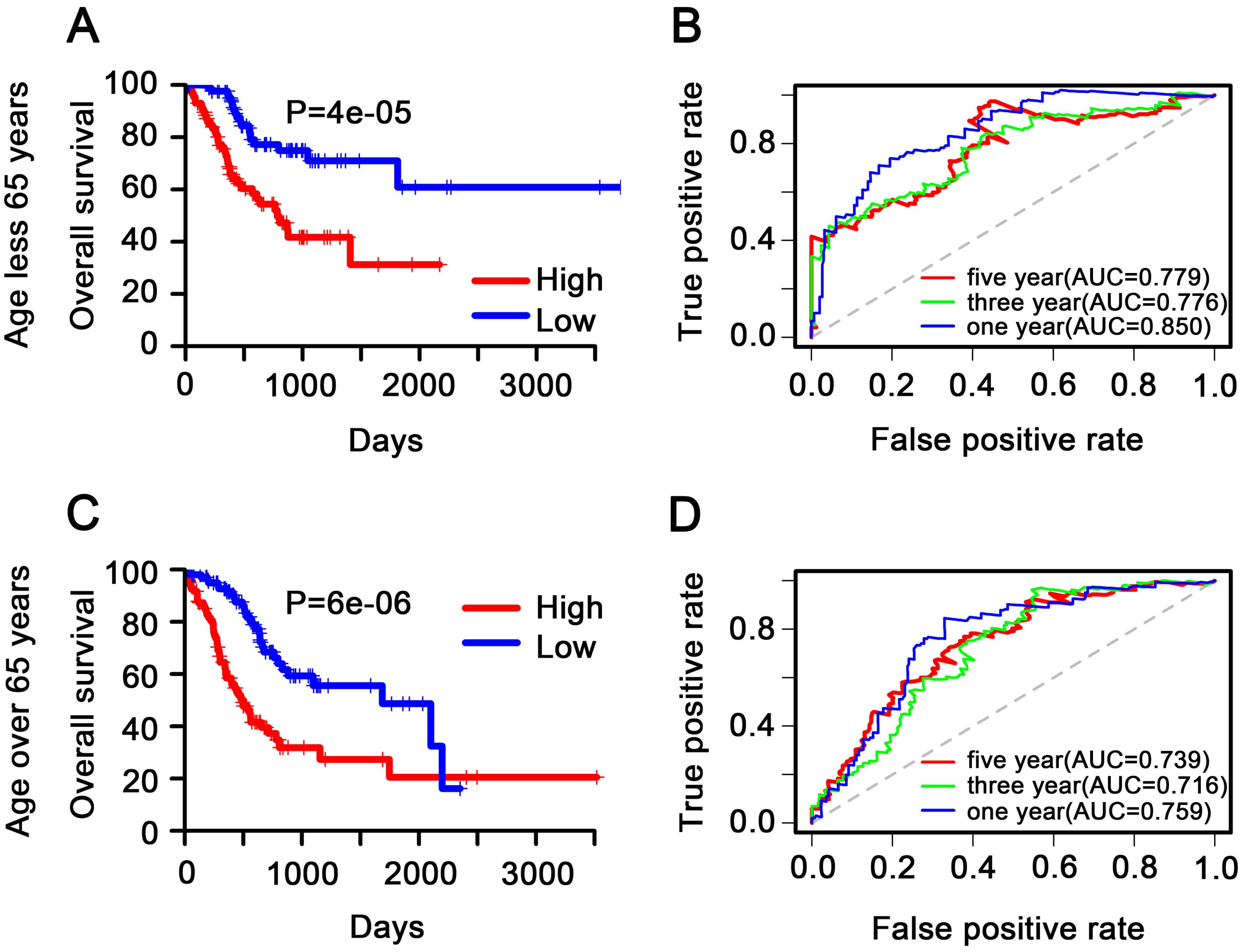

Supplementary Figure S5

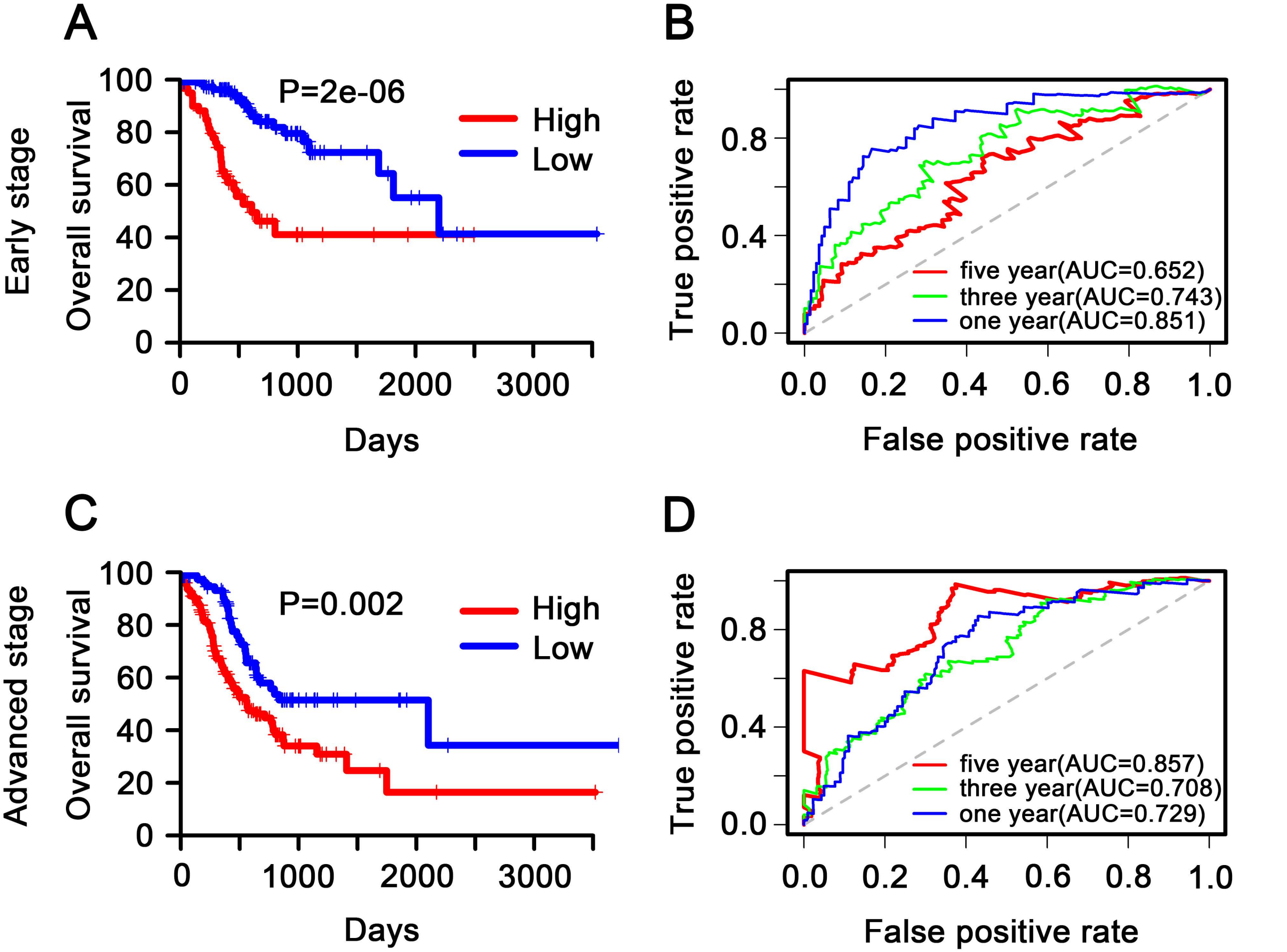

Supplementary Figure S6

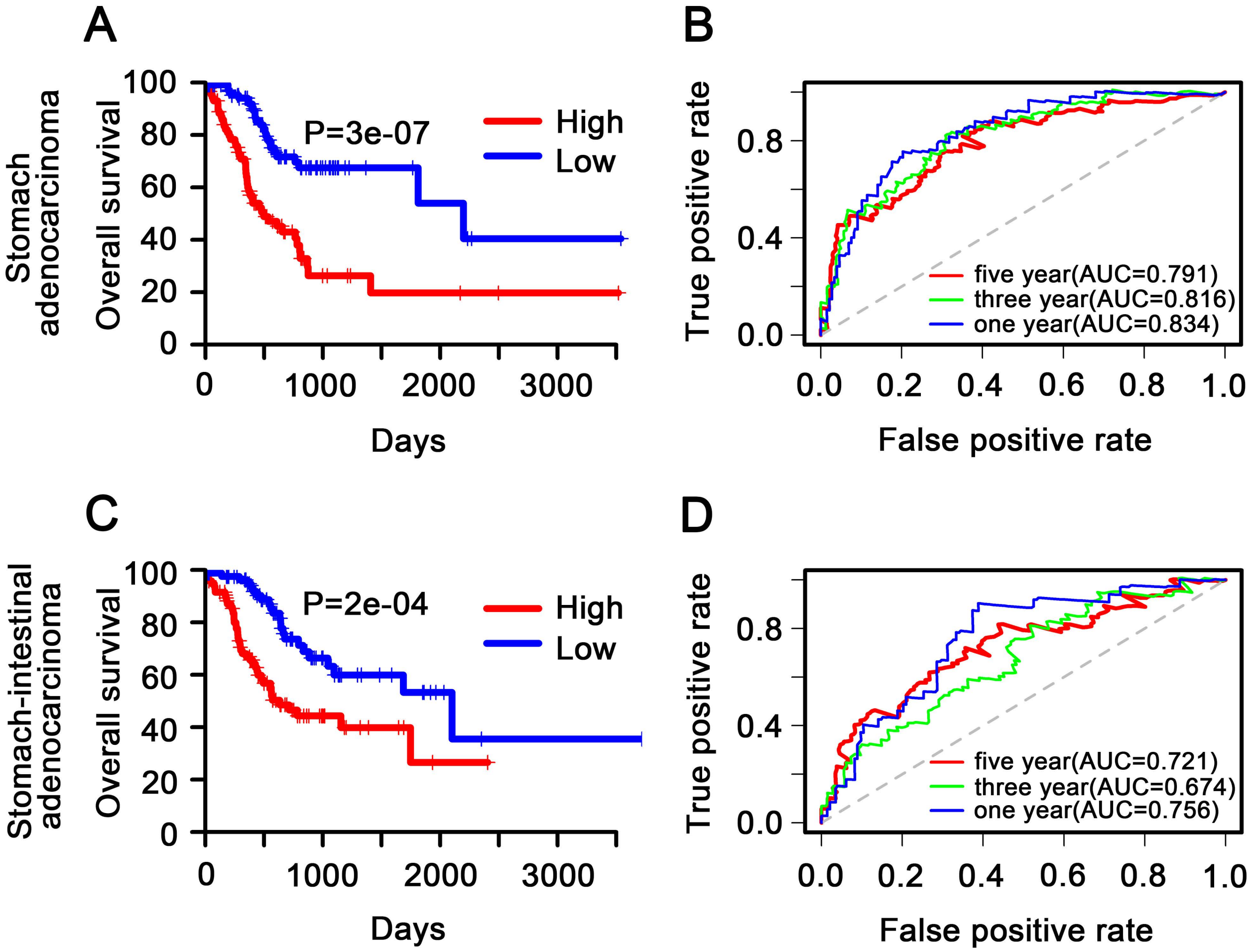

Supplementary Figure S7

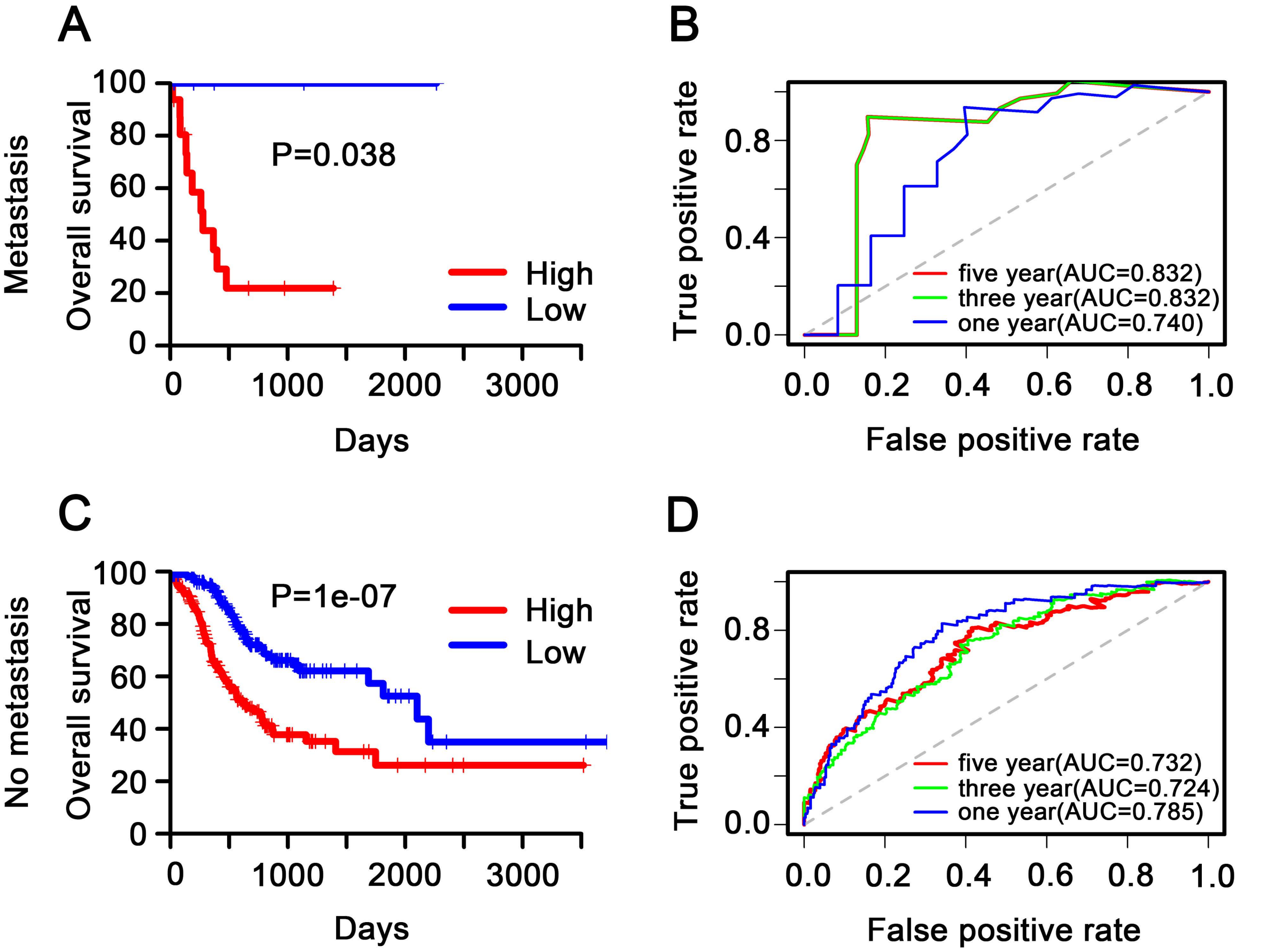

Supplementary Figure S8

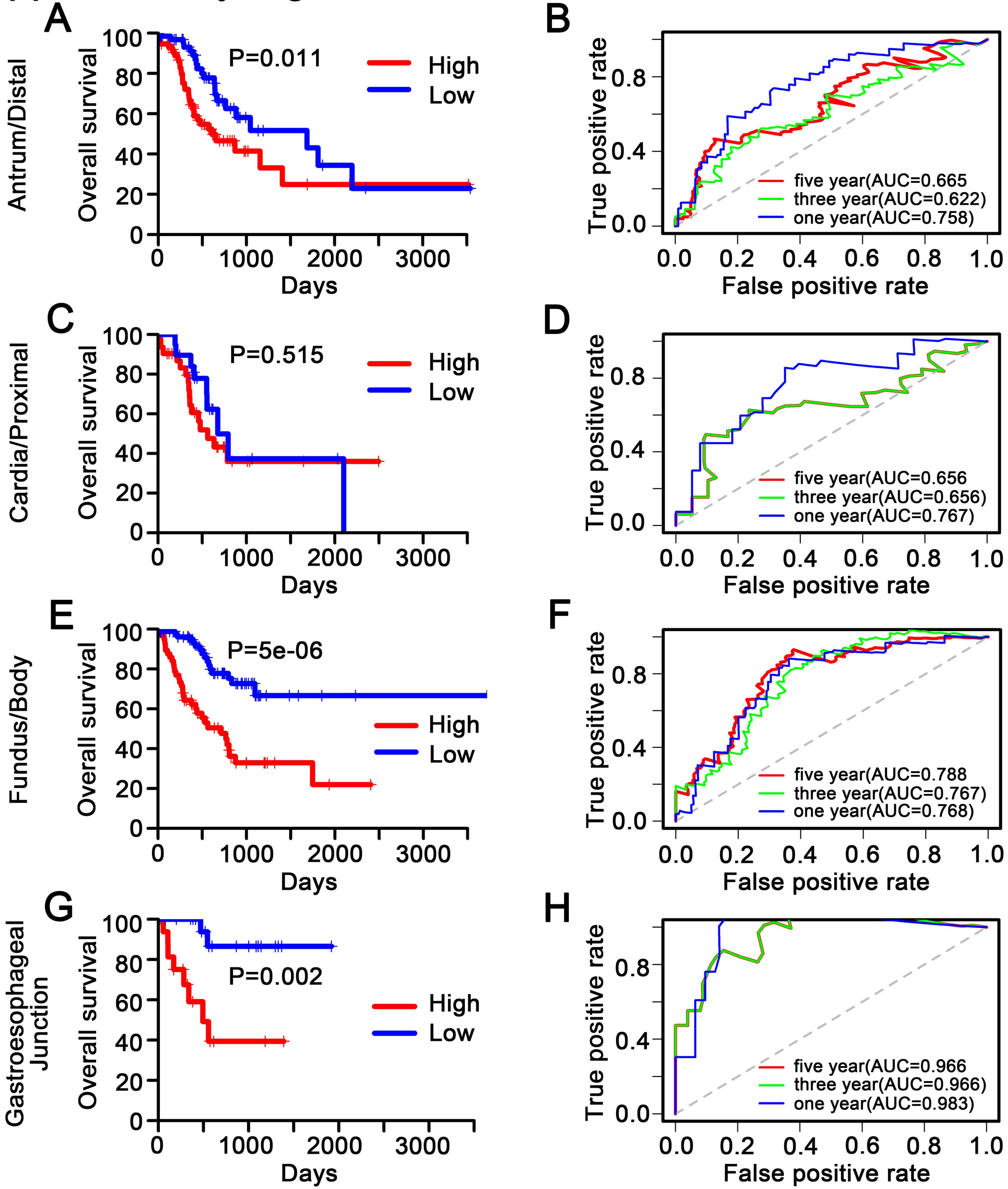

Supplementary Figure legends

Supplementary Figure S1. Boxplots of methylation β values against risk group in the training dataset.

Supplementary Figure S2. Boxplots of methylation β values against risk group in the testing dataset.

Supplementary Figure S3. Kaplan-Meier and ROC analysis of patients with STAD in sub-groups according to sex, respectively. (A, B) Female sub-group. **(C, D)** Male sub-group.

Supplementary Figure S4. Kaplan-Meier and ROC analysis of patients with STAD in sub-groups according to age, respectively. (A, B) Age less 65 years sub-group. **(C, D)** Age over 65 years sub-group.

Supplementary Figure S5. Kaplan-Meier and ROC analysis of patients with STAD in sub-groups according to stage, respectively. (A, B) Early stage sub-group. **(C, D)** Advanced stage sub-group.

Supplementary Figure S6. Kaplan-Meier and ROC analysis of patients with STAD in sub-groups according to pathological type, respectively. (A, **B)** Stomach adenocarcinoma sub-group. **(C, D)** Stomach-intestinal adenocarcinoma sub-group.

Supplementary Figure S7. Kaplan-Meier and ROC analysis of patients with STAD in sub-groups according to metastasis status, respectively. (A, B) Metastasis sub-group. **(C, D)** No metastasis sub-group.

Supplementary Figure S8. Kaplan-Meier and ROC analysis of patients with STAD in sub-groups according to anatomic site, respectively. (A, B) Antrum/Distal sub-group. (**C, D)** Cardia/Proximal sub-group. **(E, F)** Fundus/Body sub-group. **(G, H)** Gastroesophageal Junction sub-group.
